# Therapeutic Plasma Exchange and Changes in Calcium, Phosphate, Parathyroid Hormone, and Fibroblast Growth Factor-23

**DOI:** 10.1210/clinem/dgaf400

**Published:** 2025-07-15

**Authors:** Sami SeungMi Jin, Anushree Dugar, Andrew N Hoofnagle, Amber P Sanchez, David M Ward, Joachim H Ix, Charles Ginsberg

**Affiliations:** School of Medicine, University of California San Diego, 9452 Medical Center Dr, L3E206, La Jolla, CA 92037, USA; Department of Neurology, Icahn School of Medicine at Mount Sinai, New York, NY 10029, USA; Department of Laboratory Medicine and Pathology and Medicine and the Kidney Research Institute, University of Washington, Seattle, WA 98195, USA; Division of Nephrology-Hypertension, University of California San Diego, San Diego, CA 92037, USA; Division of Nephrology-Hypertension, University of California San Diego, San Diego, CA 92037, USA; Division of Nephrology-Hypertension, University of California San Diego, San Diego, CA 92037, USA; Nephrology Section, Veterans Affairs San Diego Healthcare System, San Diego, CA 92161, USA; Division of Nephrology-Hypertension, University of California San Diego, San Diego, CA 92037, USA

**Keywords:** therapeutic plasma exchange (TPE), calcium, phosphate, PTH, FGF-23

## Abstract

**Context:**

Therapeutic plasma exchange (TPE) removes plasma proteins and other unwanted substances, causing nonspecific alterations of plasma components. A previous study showed an approximately 70% reduction in vitamin D metabolites following a single TPE treatment, prompting further investigation into TPE's effects on vitamin D regulators and metabolites: calcium, phosphate, parathyroid hormone (PTH), and fibroblast growth factor-23 (FGF-23).

**Objective:**

This study examined changes in plasma and effluent levels of total calcium, phosphate, PTH, and FGF-23 in individuals undergoing TPE.

**Methods:**

Measurements were taken immediately before, immediately after, and at follow-up. Paired *t* tests compared the percentage changes in metabolites from pre- to post-TPE.

**Results:**

Study participants (N = 42) had a mean age of 55 ± 16 years, 28 (67%) were female, and 32 (76%) were White. TPE led to acute changes in calcium (−9%; 95% CI, −11% to −8%), phosphate (−14%; −18% to −11%), and FGF-23 (−12%; −18% to −6%) concentrations. In contrast, PTH levels increased (91%; 63%-119%) from baseline to post-TPE. While most metabolites returned to baseline by the follow-up visit (median 4 [interquartile range, 3-7] days), nearly 25% of patients experienced persistent asymptomatic hypocalcemia. This persistent calcium deficit was not fully corrected by continuous intravenous calcium gluconate infusions administered during the study, nor by the endogenously increased PTH levels.

**Conclusion:**

These findings underscore the need for improved care protocols and vigilant monitoring of mineral metabolism in TPE patients, especially those receiving long-term treatment. Further research is warranted to understand the enduring effects of TPE on mineral metabolism and develop strategies to prevent complications.

Therapeutic plasma exchange (TPE) is an extracorporeal treatment that involves the separation and removal of blood plasma, typically replacing it with an isotonic and iso-osmotic solution such as albumin (1). Initially developed to treat immunologic disorders by removing high-molecular-weight substances like antibodies, TPE is now widely used in the management of various autoimmune conditions (2). During this unselective removal process, other plasma components, including proteins and electrolytes, are also altered (3).

In a prior study, we demonstrated that TPE acutely reduces vitamin D–binding protein (VDBP) concentrations, thereby decreasing vitamin D metabolites such as 25-hydroxyvitamin D (25(OH)D) by approximately 70% with a single treatment (4). Given these acute and large effects on vitamin D measurements, here we investigated how TPE may affect the regulators and downstream metabolites of vitamin D activity, namely, serum total calcium, phosphate, parathyroid hormone (PTH), and fibroblast growth factor-23 (FGF-23).

Current data on metabolite removal during TPE, as well as rebound after the TPE procedure, are limited. Since our TPE procedure uses acid-citrate-dextrose solution A infusion to bind ionized calcium for regional anticoagulation in the circuit, and involves calcium gluconate infusion in the return line to avoid systemic symptomatic hypocalcemia (5), we hypothesized that calcium and PTH would remain stable while TPE would acutely decrease the concentrations of phosphate and FGF-23. As TPE is often provided recurrently over months or even years to individual patients, these changes may affect bone and mineral health in individuals receiving TPE.

## Materials and Methods

### Study Population

We have previously reported the effects of TPE on vitamin D metabolites and VDBP, as detailed in Dugar et al (3). The present study uses the same participants and stored blood samples from this cohort, which consisted of 45 patients recruited over a 3-month period, from July to September 2020, at the Apheresis Unit at the University of California, San Diego. Among these, 43 participants had undergone more than 3 TPE treatments previously, while 2 participants were enrolled during their first TPE treatment.

Participants were required to be at least age 21 years. All participants provided written informed consent, and the study was approved by the institutional review board at the University of California San Diego.

### Outcome Variables

The primary outcomes of our study were the acute changes in total corrected calcium, phosphate, PTH, and FGF-23 levels after TPE relative to the pretreatment concentrations. Samples were collected pre and post treatment, as well as at the follow-up visit. Serum total calcium and phosphate were measured using direct quantitative colorimetric determination (Stanbio Laboratory) (6). Intact parathyroid hormone (iPTH) was assessed in EDTA plasma using a 2-site immunoradiometric assay kit (N-tact PTHSP; DiaSorin). FGF-23 levels were measured using an intact assay (Kainos Laboratories). Total magnesium levels were measured in subsets of participants with available samples in May 2025. Serum albumin levels were determined using the same assay previously used for VDBP analysis, but with the inclusion of 3 albumin-specific peptides (LVNEVTEFAK, TYETTLEK, and YLYEIAR) as internal standards (3, 7).

Additionally, effluent plasma, the byproduct of the TPE, was collected during the treatment. TPE was performed on centrifugal based equipment, including the Spectra Optia (Terumo BCT) and the Amicus (Fresenius-Kabi). All patients received TPE targeting 1.0 to 1.5 times the total plasma volume, with 5% albumin as the replacement solution. Acid-citrate-dextrose solution A (3% citrate) was given via the inlet line at a ratio of 8:1 to 14:1 (whole blood to anticoagulant ratio) for regional circuit anticoagulation. Calcium gluconate 2 g in 500 mL of normal saline was given to all patients via the return line during the procedure at a rate of 80-120 mL/hour per institutional protocol (approximately 8 mEq/hour). In the subset of participants (N = 14) with available magnesium data, 50% received a mean magnesium supplementation dose of 1.4 g. Samples were stored at −80 °C until testing, ensuring the stability of all metabolites over extended periods (3, 8, 9).

To assess metabolic abnormalities, the following reference ranges were used: total calcium (8.5-10.3 mg/dL) (10), phosphate (2.5-4.5 mg/dL) (11), PTH (10-65 pg/mL) (10), and FGF-23 (19.9-52.9 pg/mL) (12). Calcium levels were corrected for albumin (13). To determine the mass (as opposed to concentration) of each metabolite removed by TPE, we assumed that the effluent volume equaled the plasma volume and calculated the respective masses in both the plasma and effluent spaces based on their concentrations and the total volume of each.

### Other Measurements

Participant demographics, comorbidities, and medication use were extracted from medical records and confirmed through participant interviews. Hypertension was defined by the prescription of blood pressure medication. Chronic kidney disease (CKD) was defined using a race-free equation, by an estimated glomerular filtration rate (eGFR) less than 60 mL/min/1.73 m^2^ at baseline (14).

### Statistical Analysis

Continuous data are described using means and SDs for variables that follow a normal distribution, and as medians and interquartile ranges (IQRs) for those with skewed distributions. Categorical data are summarized using counts and proportions. We used paired *t* tests to evaluate the unadjusted changes in each variable before and after TPE, and to compare pre-TPE values to follow-up values for assessing overall recovery. We then developed a sequence of models using multiple linear regression to determine if any of observed changes were influenced by confounding factors. Model 1, our primary analytic model, was adjusted for age, sex, race, body mass index (BMI), eGFR, TPE indication, TPE duration of a single procedure, days between TPE procedures, number of TPE procedures performed before sample collection, and the weekly dose supplementation of calcium, iron, and phosphate. Model 2 was additionally adjusted for percentage changes in metabolites. All analyses were performed using Stata SE v.15.1. Statistical significance was defined as *P* values less than .05 for all tests.

## Results

We recruited 45 participants to this study. Two individuals were excluded because of missing FGF-23 data. Additionally, one person who received a mixture of half albumin and half plasma replacement was excluded. The baseline characteristics of the remaining 42 participants that served as the study cohort are depicted in Table 1. The mean age was 55 ± 16 years, with a mean BMI of 25.7 ± 5.6. Sixty-seven percent were female, 76% were White, and 40% had been previously diagnosed with osteopenia or osteoporosis. Additionally, 19% had experienced at least 1 bone fracture within the past 5 years, and 2 participants had CKD, including 1 who had a kidney transplant. The most common indication for TPE was multiple sclerosis (48%), followed by myasthenia gravis (17%) and autoimmune autonomic neuropathy (14%). The median (IQR) number of TPE procedures performed before sample collection was 127 (52-418), with each procedure lasting on average 105 ± 23 minutes. The median (IQR) time in days between the index procedure and the follow-up blood measurements was 4 (3-7). The baseline median (IQR) concentration of total corrected calcium was 9.4 mg/dL (9.2-9.6), phosphate was 3.6 mg/dL (3.3-3.9), PTH was 43 pg/mL (35-59), and iFGF-23 was 42 pg/mL (32-55).

**Table 1.** Baseline characteristics of therapeutic plasma exchange patients (N = 42)

Variables	
Age ± SD, y	55 ± 16
Female sex, n (%)	28 (67)
Race, n (%)	
White	32 (76)
Asian	2 (5)
Black	2 (5)
Other	6 (14)
eGFR ± SD, mL/min/1.73 m^2^	96.6 ± 15.9
BMI ± SD	25.7 ± 5.6
Treatment parameters	
No. of treatments (IQR)	127 (52-418)
Duration, min	105 ± 23
Days between treatments (IQR)	4 (3-7)
Plasma removed, mL	3057 ± 619
Fluid replaced, mL	3459 ± 672
TPE indications, n (%)	
MS	20 (48)
MG	7 (17)
Autoimmune autonomic neuropathy	6 (14)
CIDP	3 (7)
Comorbidities, n (%)	
Hypertension	13 (31)
Bone fracture in the past 5 y	8 (19)
CKD	2 (5)
Renal transplant	1 (2)
Liver disease	1 (2)
Pretreatment biochemistry	
Total corrected calcium, median (IQR), mg/dL	9.4 (9.2-9.6)
Phosphate, median (IQR), mg/dL	3.6 (3.3-3.9)
Parathyroid hormone, median (IQR), pg/mL	43 (35-59)
iFGF-23, median (IQR), pg/mL	42 (32-55)
25-Hydroxyvitamin D, median (IQR), ng/mL	44 (34-57)
1,25-Dihydroxyvitamin D, median (IQR), ng/mL	48 (39-62)
Magnesium, median (IQR), mg/dL*^a^*	1.9 (1.8-2.1)
Albumin, g/dL	4.7 (4.5-4.9)
Supplementation	
Calcium, n (%)	18 (43)
Weekly mg (IQR)	0 (0-1540)
Phosphate, n (%)	12 (29)
Weekly IU (IQR)	0 (0-140)
Ergocalciferol, n (%)	13 (31)
Weekly IU (IQR)	0 (0-50 000)
Cholecalciferol, n (%)	32 (76)
Weekly IU (IQR)	21 000 (7000-37 800)
Iron, n (%)	16 (38)
Weekly IU (IQR)	0 (0-126)

eGFR was calculated using the 2021 CKD–Epidemiology Collaboration serum creatinine equation.

Abbreviations: BMI, body mass index; CIPD, chronic inflammatory demyelinating polyneuropathy; CKD, chronic kidney disease; eGFR, estimated glomerular filtration rate; iFGF-23, intact fibroblast growth factor-23; IQR, interquartile range; IU, international units; MG, myasthenia gravis; MS, multiple sclerosis; TPE, therapeutic plasma exchange.

^*a*^Magnesium levels were measured in a subset of participants (n = 14) with available samples.

### Changes of Mineral Parameters From Before to After a Single Therapeutic Plasma Exchange Procedure

**Figure 1.**
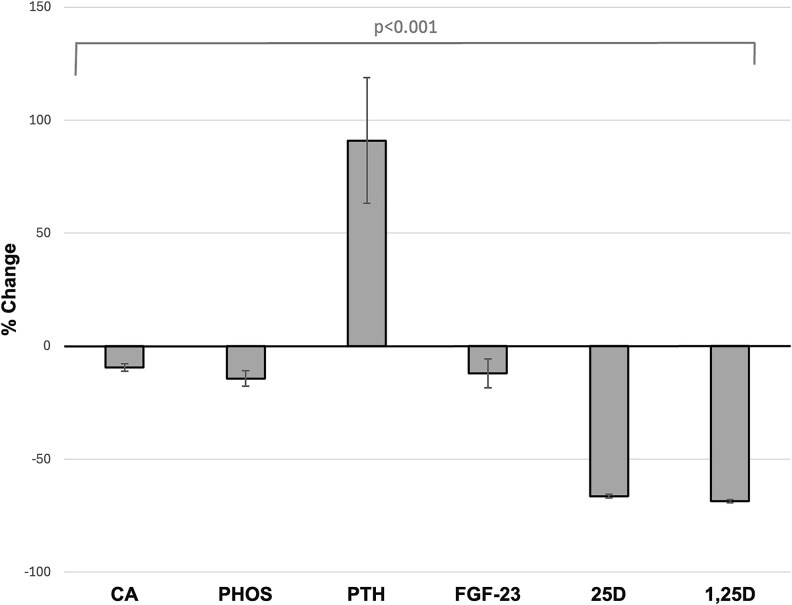
Percentage changes in total corrected calcium, phosphate, PTH, FGF-23, 25D, and 1,25D from pre– to post–therapeutic plasma exchange (TPE) (N = 42). Acute reductions in all metabolites, except for an increase in PTH.

Abbreviations: CA, calcium; PHOS, phosphate; PTH, parathyroid hormone; FGF-23, fibroblast growth factor 23; 1,25D, 1,25-dihydroxyvitamin D; 25D, 25-hydroxyvitamin D.

**Figure 2.**
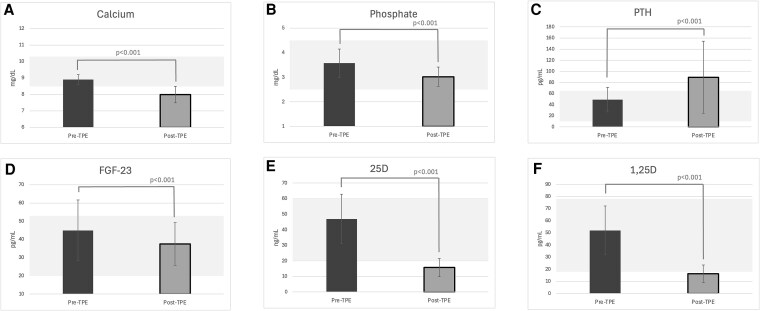
A to F, Comparison of metabolite levels (mean ± SD) from pre– to post–therapeutic plasma exchange (TPE) for A, calcium; B, phosphate; C, PTH; D, FGF-23; E, 25D; and F, 1,25D, respectively (N = 42). The gray shaded areas indicate reference ranges for each metabolite: calcium (8.5-10.3 mg/dL), phosphate (2.5-4.5 mg/dL), PTH (10-65 pg/mL), FGF-23 (19.9-52.9 pg/mL), 25D (20-60 ng/mL), 1,25D (18-78 pg/mL). All observed changes were statistically significant (*P* < .001).

Abbreviations: 1,25D, 1,25-dihydroxyvitamin D; 25D, 25-hydroxyvitamin D; FGF-23, fibroblast growth factor 23; PTH, parathyroid hormone.

**Figure 3.**
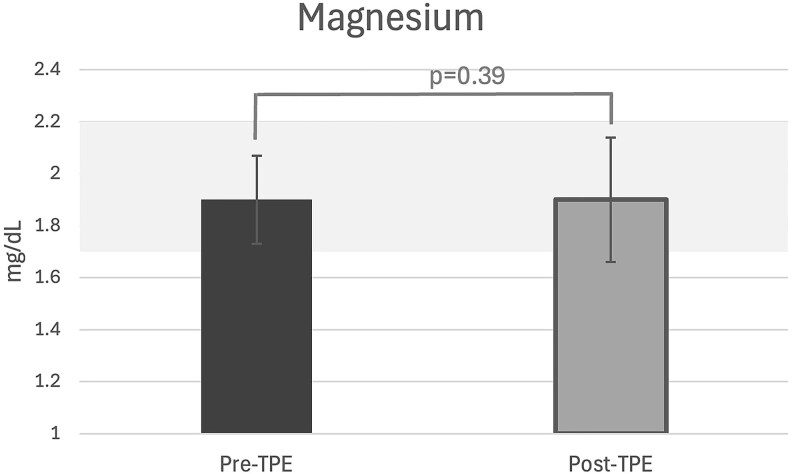
Magnesium levels before and after a single therapeutic plasma exchange (TPE) procedure. Pre-TPE (n = 14); post-TPE (n = 13). Gray shaded area indicates reference range 1.7 to 2.2 mg/dL. No statistically significant difference was observed (*P* = .39).

In unadjusted models, TPE caused a statistically significant change in total corrected calcium of −9% (95% CI, −11% to −8%), phosphate −14% (−18% to −11%), iFGF-23 −12% (−18% to −6%), 25D −66% (−68 to −65), and 1,25D −69% (−70 to −67) (*P* < .001 for all). Concurrently, we observed a significant and marked increase in PTH of 91% (63%-119%) compared to baseline (*P* < .001) (Fig. 1). In model 1, each year older age was associated with lesser reductions in total corrected calcium +0.18% (95% CI, 0.003%-0.35%) and each unit mL/min/1.73 m^2^ higher eGFR was also associated with lesser reductions in total corrected calcium +0.2% (0.0%-0.3%) (Table 2). An increase of 1 minute in TPE duration was associated with a 0.3% (0.2%-0.3%) lesser reduction in total corrected calcium. In model 2, which accounted for the changes in the other mineral metabolism measures concurrently, a 10% increase in PTH during TPE was associated with a −0.2% (−0.3%-0.0%) concurrent change in total corrected calcium (*P* = .03). Fig. 2 depicts the mean changes in the aforementioned metabolites, as well as vitamin D metabolites, from before to after a single TPE procedure, with reference ranges indicated for each metabolite (*P* < .001 for all). Total magnesium, shown separately in Fig. 3 for participants with available data, did not change significantly during the same period (*P* = .39). However, there was a significantly positive association between the percentage change in magnesium levels and the dose of supplementation: for each 1-gram increase in magnesium supplementation, there was a 16.3% increase in magnesium levels from pre to post TPE.

**Table 2.** Association of patient and therapeutic plasma exchange (TPE) parameters with serum total corrected calcium changes during TPE among 42 patients

Variables	Model 1		Model 2	
	% change in total calcium (95% CI)	*P*	% change in total calcium (95% CI)	*P*
Age, y	0.2 (0.0 to 0.3)	.034	0.2 (0.0 to 0.3)	.02
Sex	4 (−1 to 98)	.05	3 (0 to 7)	.06
TPE indication*		.004		.07
CIDP	−12 (−27 to −7)	<.001	−9 (−18 to −5)	.001
MG	−5 (−8 to −2	.01	−3 (−6 to −1)	.02
eGFR	0.2 (0.0 to 0.3)	.01	0.1 (0.0 to 0.2)	.01
Supplementation				
Weekly total phos	0.002 (0.000 to 0.004)	.045	0.002 (0.001 to 0.004)	.01
Weekly total iron	−0.002 (−0.003 to −0.001)	.01	−0.001 (−0.002 to −0.000)	.06
Duration, min	0.3 (0.2 to 0.4)	<.001	0.2 (0.1 to 0.3)	.001
Days between treatments	0.3 (−0.0 to 0.7)	.06	0.3 (0.1 to 0.6)	.02
No. of treatments	0 (0 to 0)	.02	0 (0 to 0)	.03
% change per 10% change of metabolites				
Phosphate			1.12 (0.23 to 2.13)	.02
PTH			−0.2 (−0.3 to −0.0)	.02
iFGF-23			0.2 (−0.2 to 0.7)	.26

Model 1 was adjusted for age, sex, race, BMI, eGFR, TPE indication, duration, days between TPE procedures, number of TPE procedures performed before sample collection, and the weekly dose supplementation of calcium, iron, and phosphate.

Model 2 was additionally adjusted for percentage changes in metabolites.

Abbreviations: BMI, body mass index; CIDP, chronic inflammatory demyelinating polyneuropathy; eGFR, estimated glomerular filtration rate; iFGF-23, intact fibroblast growth factor-23; MG, myasthenia gravis; PTH, parathyroid hormone.

^*^Multiple sclerosis serves as reference category.

### Mass Removal of Metabolites

The median (IQR) concentration of metabolites in the effluent fluid was: total calcium 6.9 mg/dL (6.7-7.2); phosphate, 2.7 mg/dL (2.5-3); PTH, 58 pg/mL (42-80); iFGF-23, 48 pg/mL (38-54); 25D, 26.0 ng/mL (18.3-31.3); 1,25D, 26.8 ng/mL (20.0-31.5); total magnesium, 1.9 mg/dL (1.7-2.1) (Table 3). The percentage mass removed for each metabolite, calculated relative to its estimated initial plasma mass and assuming effluent volume was equal to plasma volume, was as follows: total calcium, 74% (95% CI, 72%-75%); phosphate, 77% (74%-79%); PTH, 143% (127%-161%); iFGF-23, 110% (103%-117%); 25D, 56% (52%-59%); 1,25D, 53% (50%-55%); and magnesium, 95% (84%-105%).

**Table 3.** Effluent concentrations and percentage removed by therapeutic plasma exchange

	Concentration, median (IQR)	Estimated % removed, mean (95%CI)
Calcium	6.9 mg/dL (6.7-7.2)	74% (72%-75%)
Phosphate	2.7 mg/dL (2.5-3)	77% (74%-79%)
PTH	58 pg/mL (42-80)	143% (127%-161%)
iFGF-23	48 pg/mL (38-54)	110% (103%-117%)
25D	26.0 ng/mL (18.3-31.3)	56% (52%-59%)
1,25D	26.8 µg/mL (20.0-31.5)	53% (50%-55%)
Magnesium*^a^*	1.9 mg/dL (1.7-2.1)	95% (84%-105%)

Calculation assumes plasma volume equals effluent volume.

Abbreviations: 1,25D, 1,25-dihydroxyvitamin D; 25D, 25-hydroxyvitamin D; iFGF-23, intact fibroblast growth factor-23; IQR, interquartile range; PTH, parathyroid hormone.

^*a*^Magnesium levels were measured in a subset of participants (n = 12) with available samples.

### Metabolite Abnormalities

Thirty-five participants (83%) were hypocalcemic when defined as total corrected calcium levels below the lower limit of normal (total calcium <8.5 mg/dL) at post TPE. Most participants had recovery of total calcium by the follow-up visit, but 10 participants (24%) remained hypocalcemic at follow-up (Table 4). Four participants (10%) were hypophosphatemic at post TPE. Most recovered to normal phosphate levels at the follow-up visit, with one participant (2%) remaining hypophosphatemic. Twenty-seven participants (64%) were hyperparathyroid post TPE, with 7 participants (17%) remaining hyperparathyroid at follow-up. No participants experienced hypercalcemia or hypoparathyroidism at any point during the study. Three participants (7%) had low iFGF-23 levels at post TPE, and 2 participants (5%) still had low iFGF-23 at follow-up. Total magnesium levels were measured in subsets of participants with available data. Four of 13 participants (31%) at post TPE and 1 of 10 participants (10%) at follow-up had mildly low magnesium levels, all measuring 1.6 mg/dL (Table 5).

**Table 4.** Metabolite abnormalities among 42 therapeutic plasma exchange participants

Variable	No. of participants (%)		
	Pre-TPE	Post-TPE	Follow-up
Hypocalcemia	6 (14)	35 (83)	10 (24)
Hypercalcemia	0 (0)	0 (0)	0 (0)
Hypophosphatemia	1 (2)	4 (10)	1 (2)
Hyperphosphatemia	3 (7)	0 (0)	0 (0)
Hypoparathyroidism	0 (0)	0 (0)	0 (0)
Hyperparathyroidism	9 (21)	27 (64)	7 (17)
Low iFGF-23	2 (5)	3 (7)	2 (5)
High iFGF-23	12 (29)	3 (7)	6 (14)

Reference values: calcium (8.5-10.3 mg/dL), phosphate (2.5-4.5 mg/dL), PTH (10-65 pg/mL), and FGF-23 (19.9-52.9 pg/mL).

Calcium levels were corrected for albumin.

Abbreviations: FGF-23, fibroblast growth factor-23; iFGF-23, intact fibroblast growth factor-23; PTH, parathyroid hormone; TPE, therapeutic plasma exchange.

**Table 5.** Magnesium abnormalities across therapeutic plasma exchange procedure

Variable	No. of participants (%)		
	Pre-TPE n = 14	Post-TPE n = 13	Follow-up n = 10
Hypomagnesemia*^a^*	1 (7)	4 (31)	1 (10)
Hypermagnesemia*^a^*	0 (0)	1 (8)	0 (0)

Reference range: 1.7 to 2.2 mg/dL.

Abbreviation: TPE, therapeutic plasma exchange.

^*a*^Magnesium levels were measured in subsets of participants with available samples.

## Discussion

This study examined the acute effect of TPE on the key regulators and metabolites of vitamin D: calcium, phosphate, PTH, and FGF-23. Levels of these metabolites were measured pre TPE, immediately post TPE, and at a follow-up visit. Immediately post TPE, we observed a significant decrease in total calcium, phosphate, and FGF-23 levels, while PTH increased significantly and substantially. For all metabolites except total calcium, these changes appeared transient, as most had normalized by the follow-up visit. On the other hand, 24% of participants remained hypocalcemic at the follow-up visit. This sustained reduction suggests potential long-term effects of TPE on mineral metabolism, warranting further investigation and possible changes in management, particularly for patients who remain on TPE for a prolonged course.

The acute decline in calcium, phosphate, and FGF-23 likely resulted from direct metabolite clearance during TPE and potential dilution from albumin infusions. During TPE, a colloid-containing replacement fluid, typically albumin, is infused to prevent hypotension and edema (15). While iso-oncotic albumin solutions are commonly used to maintain plasma volume, it poses some risks such as plasma dilutions. Hedin and Hahn demonstrated that the 5% albumin infusion expanded plasma volume by 75% of the infused volume, presumably due to recruitment from the extravascular space with a maximum plasma dilution of 17% (16). These findings suggest that 5% albumin infusions administered during the 105 ± 23 minutes of the TPE procedure may have significantly diluted the plasma, leading to reductions in the concentrations of plasma constituents, including the metabolites of interest in our study. However, these findings may not be applicable to patients undergoing TPE as they are concurrently having albumin directly removed, in a close to 1:1 ratio of infusion.

PTH increased acutely, which was unexpected given its substantial clearance of 143% (95% CI, 127%-161%) in the effluent fluid. Notably, our removal estimates are conservative, as they assume that plasma volume equals effluent volume. In practice, however, the effluent volume is typically 100% to 150% greater than the plasma volume (17). The rapid surge in PTH was likely a compensatory response to hypocalcemia, reflecting the body's immediate attempt to maintain calcium homeostasis. This response may have been further amplified by reduced calcitriol (3) and FGF-23 during TPE, both of which normally suppress PTH (18). Our previous study showed significant reductions both in 25D and 1,25D—metabolites that inhibit PTH directly as well as indirectly by promoting intestinal calcium and phosphate absorption. During treatment, diminished direct suppression appears to have contributed to the PTH surge. From treatment to follow-up, the lack of indirect suppression due to reduced calcium and phosphate absorption appears linked to the worsened hypocalcemia observed in 24% of patients at follow-up and sustained PTH elevation at 17%.

We also explored the role of magnesium in PTH regulation. Although no significant change in total magnesium levels was observed from pre to post TPE (see Fig. 3), there was a significantly positive association between the percentage change in magnesium levels and the dose of supplementation. Specifically, each additional gram of magnesium administered was associated with a 16.3% increase in magnesium levels from pre to post TPE. Considering the high 95% clearance of circulating magnesium during TPE, these findings suggest that supplementation mitigated a more substantial decline in magnesium levels. Despite supplementation, 4 participants were mildly hypomagnesemic (all with levels of 1.6 mg/dL) at post TPE, and 1 remained hypomagnesemic at follow-up (also 1.6 mg/dL) (see Table 5). While mild hypomagnesemia can stimulate PTH production, severe reductions in serum magnesium are known to suppress PTH (19). The relatively stable magnesium levels achieved through supplementation argue against it being the main driver of the observed PTH surge. Instead, as discussed earlier, hypocalcemia appears to be the main contributor. Additionally, without magnesium supplementation, the PTH response may have been blunted due to large declines in magnesium, possibly leading to worsened hypocalcemia.

Importantly, the observed episodes of hypocalcemia, despite the marked rise in PTH, suggests that the compensatory mechanism was insufficient to counteract ongoing calcium depletion. Several factors likely contributed to the significant calcium loss: the infusion of calcium-avid albumin replacement solution, the chelation and removal of citrate-bound calcium, and the direct removal of all calcium-containing complexes. The calcium-avid nature of the albumin replacement solution may have further depleted the serum ionized calcium, which was already reduced by citrate chelation (20). Additionally, the removal of both calcium-bound albumin and citrate may have further diminished the total calcium pool, worsening the calcium deficit (20). Collectively, these processes likely contributed to a more severe calcium deficit that failed to be adequately corrected by endogenous PTH or the calcium gluconate infusions administered in this study.

To counteract citrate-induced hypocalcemia during TPE, calcium supplementation is commonly administered, though practices vary across institutions. Some centers provide no calcium unless hypocalcemic symptoms arise. At our center, every patient receiving citrate-anticoagulated TPE has received a continuous intravenous infusion of calcium to the return line, in dilute concentration (calcium gluconate or calcium chloride adjusted to a concentration of 1 mEq per 10 mL, at a rate usually ∼8 meq/hour). This consistent supplementation may explain the lower incidence of clinically overt hypocalcemia and the relatively stable levels of calcium and other measured parameters, compared to studies in which calcium is not supplemented (5, 21). Despite this precaution, we observed episodes of persistent asymptomatic hypocalcemia (24%) and hyperparathyroidism (17%) in patients 3 to 7 days later. While no study participants experienced overt symptoms of hypocalcemia, such as muscle cramping, headaches, and chest discomfort (5, 22), the observed hypocalcemia and hyperparathyroidism, without concurrent hypercalcemia or hypoparathyroidism, suggest that our current calcium-replacement protocol may be insufficient for many individuals. Elevated PTH levels raise concerns about bone demineralization, emphasizing the need for careful monitoring when prolonged TPE courses are anticipated (10). Chronic TPE patients may benefit from dual-energy x-ray absorptiometry scans to assess for boss loss, perhaps at baseline and again after 2 years (23). Incorporating bone turnover markers such as C-terminal telopeptide, N-terminal telopeptide, and tartrate-resistant acid phosphatase 5b could facilitate early detection of bone-remodeling changes and guide timely interventions (24).

Optimizing calcium homeostasis requires a multifaceted approach. Potential strategies may include increasing the infusion rate, using a more potent calcium supplement, or exploring alternative strategies like substituting the replacement solution with a calcium-neutral colloid (25-27). Adjunctive therapies, including vitamin D and calcitriol, may also help, given the reductions in these metabolites observed during treatment (3). Notably, free vitamin D levels remained relatively stable (3), suggesting that targeted supplementation could enhance calcium and PTH regulation. This approach may be particularly relevant for patients undergoing TPE for autoimmune conditions, as many receive prednisone, a known inhibitor of vitamin D activation (28). In such cases, calcitriol supplementation may help reduce the potential for prednisone-induced metabolite dysfunction, independent of TPE-related effects.

Interestingly, a 1-minute increase in TPE duration was associated with a 0.3% (95% CI, 0.2%-0.3%) smaller reduction in total corrected calcium, suggesting that extending the duration of TPE procedures may allow for more effective release of calcium from bone via PTH. Together, these findings suggest that increasing the calcium infusion rates, combined with other strategies to reduce calcium depletion, may be beneficial in preventing or mitigating hypocalcemia in our patients.

FGF-23 primarily regulates phosphate homeostasis, increasing in response to elevated serum phosphate levels and decreasing when phosphate levels fall (22). In our study, we observed a decrease both in iFGF-23 and phosphate. While hypophosphatemia has known adverse effects, including muscle cramping, weakness, respiratory depression, rhabdomyolysis, and osteomalacia (29), the clinical implications of low FGF-23 remain unclear. Despite significant total mass removal of iFGF-23 (based on effluent concentrations) by TPE (110%; 95% CI, 103%-117%) and reduction of phosphate, plasma iFGF-23 concentrations changed by only −12% (95% CI, −18% to −6%). This observation may be due to several factors. First, FGF-23 has a relatively short half-life of 46 to 58 minutes (30), while the average duration of the TPE procedure in this study was 105 ± 23 minutes. Due to its rapid clearance from the bloodstream, FGF-23 concentrations may be less affected by the procedure compared to substances with longer half-lives (31). Second, an acute increase in PTH, potentially triggered by transient hypocalcemia during TPE, may have stimulated rapid FGF-23 production (18, 32). However, the interpretations are complicated by the limitations of the second-generation DiaSorin N-tact PTH assay, which, although designed to measure iPTH, also detects some inactive C-terminal PTH fragments (33). The potential overestimation of PTH levels may partially explain the relatively modest changes in phosphate levels and consequently, the limited reduction in FGF-23. Although FGF-23 kinetics are not fully understood, a study by Durlacher-Betzer et al (34) demonstrated that folic-acid induced acute kidney injury leads to a 5-fold increase both in FGF-23 messenger RNA and serum iFGF-23 levels within just 3 hours in murine models. However, it remains unclear whether this increase in iFGF-23 is all generated from the new messenger RNA synthesis or if preformed vesicles within cells or an extracellular reservoir of iFGF-23 could contribute to the rapid response. Last, the reduction in phosphate may not have led to any changes in iFGF-23 within the short duration of TPE. iFGF-23 levels typically peak 8 to 12 hours after phosphate administration (35). This delay between phosphate changes and iFGF-23 response could explain why the observed decrease in iFGF-23 was smaller than anticipated during TPE, despite the reduction in phosphate. In summary, the smaller-than-expected reduction in iFGF-23, despite its significant removal by TPE, may be due to the interplay between iFGF-23's inherent characteristics and potential stimulation or lack thereof by related metabolites. This underscores the need for further research into the mechanisms of FGF-23 secretion and physiologic storage to better understand its regulation.

The strengths of this study include the repeated measurement of metabolites at 3 distinct time points and the collection of effluent fluid. This approach allowed for the determination of the mass and proportion of each metabolite removed during TPE, confirming their removal and facilitating the analysis of other factors contributing to changes in metabolite levels. By assessing both the percentage changes and the removal data, we were able to gain a better understanding of the metabolite dynamics during TPE. This study also has several important limitations. The relatively small study population may limit the generalizability of the findings. Additionally, the absence of an external control group limits the ability to compare findings beyond our intervention. Nevertheless, we believe that the pre-TPE levels serve as a robust internal control for each participant. Finally, this study was underpowered to detect potential effects of different anticoagulant ratios on the changes in total calcium and PTH, and further work is needed to explore this end point.

In summary, TPE induced acute metabolite changes in all metabolites evaluated here. Most of these changes subsequently normalized within 3 to 7 days. In contrast, despite using calcium infusions in the return line in all participants, hypocalcemia was common and was sustained at the follow-up visit in nearly one-fourth of participants. Marked acute increases in PTH were observed during TPE, likely in an effort to protect against acute hypocalcemia. These findings highlight the importance of careful monitoring during and after TPE to mitigate potential adverse effects and ensure patient safety, and argue for routine use of calcium infusions in the return line in most patients receiving TPE. Individuals on long-term TPE may have substantial bone loss if calcium and PTH concentrations are not adequately monitored.

## Data Availability

The data underlying this article will be shared on reasonable request to the corresponding author.

## Abbreviations

1,25D: 1,25-dihydroxyvitamin D
25D: 25-hydroxyvitamin D
BMI: body mass index
CKD: chronic kidney disease
eGFR: estimated glomerular filtration rate
FGF-23: fibroblast growth factor-23
iFGF-23: intact fibroblast growth factor-23
iPTH: intact parathyroid hormone
IQR: interquartile range
PTH: parathyroid hormone
TPE: therapeutic plasma exchange
VDBP: vitamin D–binding protein
